# Supplementary material for: Causal associations between type 1 diabetes mellitus and cardiovascular diseases: a Mendelian randomization study
Source: Cardiovasc Diabetol. 2023 Sep 2;22:236. doi: 10.1186/s12933-023-01974-6 (PMC10475187; doi:10.1186/s12933-023-01974-6)
Supplement: Supplementary file 1 — Supplementary Material 1 [file 12933_2023_1974_MOESM1_ESM.docx]

***Supplementary Materials***

**Causal associations between type 1 diabetes mellitus and cardiovascular diseases: A mendelian randomization study**

Zirui Liu, Haocheng Wang, Zhengkai Yang, Yu Lu, Cao Zou

**Contents**

**Figure S1.** Study design and 3 assumptions of MR analysis.

**Table S1.** Causal effects of CVDs on T1DM.

**Table S2.** Validation analysis on the causal effects of T1DM on CVDs in UK-biobank.


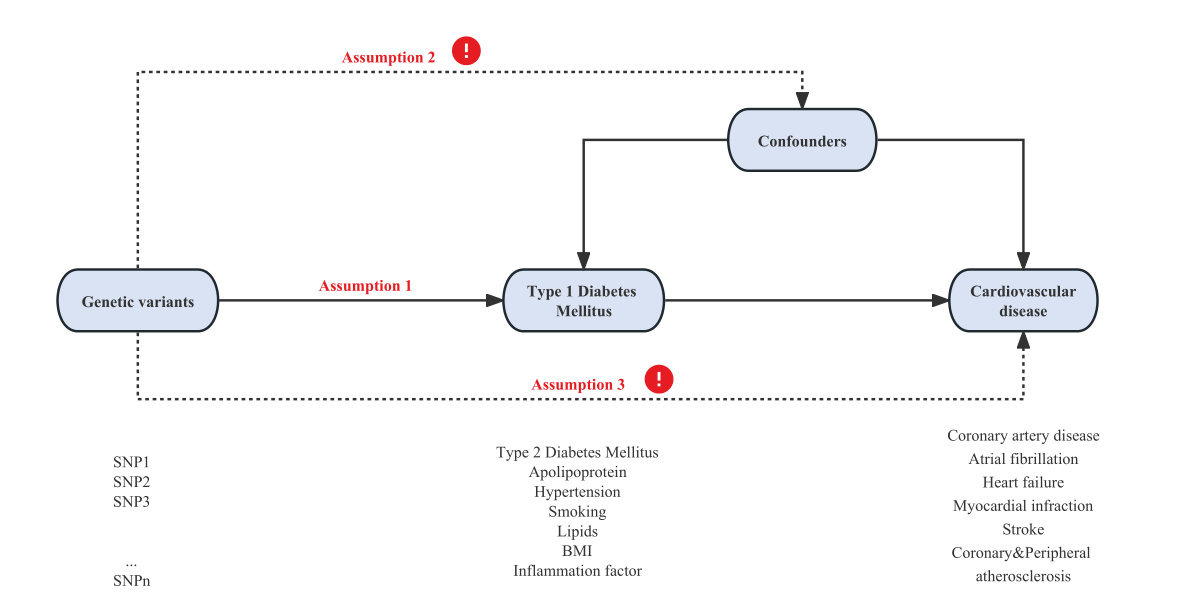


**Figure S1.** Study design and 3 assumptions of MR analysis.

**Table S1.** Causal effects of CVDs on T1DM.

| **Exposure** | **Method** | **nsnp** | **Pval** | **OR** | **95%CI** |
| --- | --- | --- | --- | --- | --- |
| Coronary artery disease | Inverse variance weighted | 62 | 0.53 | 1.04 | 0.92-1.17 |
|  | Weighted median |  | 0.66 | 0.97 | 0.83-1.13 |
|  | MR Egger |  | 0.38 | 1.11 | 0.88-1.41 |
| Atrial fibrillation | Inverse variance weighted | 111 | 0.59 | 0.98 | 0.91-1.05 |
|  | Weighted median |  | 0.25 | 0.93 | 0.83-1.05 |
|  | MR Egger |  | 0.32 | 1.08 | 0.93-1.24 |
| Stroke | Inverse variance weighted | 8 | 0.37 | 1.65 | 0.55-4.93 |
|  | Weighted median |  | 0.21 | 0.76 | 0.49-1.17 |
|  | MR Egger |  | 0.84 | 0.40 | 0-1657.37 |
| Heart failure | Inverse variance weighted | 9 | 0.21 | 0.79 | 0.55-1.14 |
|  | Weighted median |  | 0.11 | 0.72 | 0.48-1.07 |
|  | MR Egger |  | 0.48 | 0.65 | 0.22-1.98 |
| Myocardial infarction | Inverse variance weighted | 78 | 0.35 | 1.07 | 0.93-1.24 |
|  | Weighted median |  | 0.69 | 0.97 | 0.84-1.12 |
|  | MR Egger |  | 0.44 | 1.13 | 0.83-1.55 |
| Peripheral atherosclerosis | Inverse variance weighted | 6 | 0.26 | 2.66 | 0.49-14.40 |
|  | Weighted median |  | 0.97 | 1.00 | 0.83-1.21 |
|  | MR Egger |  | 0.62 | 0.13 | 0-251.40 |
| Coronary atherosclerosis | Inverse variance weighted | 30 | 0.35 | 1.21 | 0.81-1.80 |
|  | Weighted median |  | 0.81 | 0.98 | 0.86-1.12 |
|  | MR Egger |  | 0.60 | 0.78 | 0.31-1.95 |

**Table S2.** Validation analysis on the causal effects of T1DM on CVDs in UK-biobank.

| **Exposure** | **Outcome** | **Method** | **nsnp** | **Pval** | **OR(95%CI)** | **MR_egger_P** | **MR_PRESSO_P** |
| --- | --- | --- | --- | --- | --- | --- | --- |
| T1DM (ebi-a-GCST010681) | Coronary atherosclerosis  (I9_CORATHER) | Inverse variance weighted | 24 | 0.65 | 1.0002  (0.9995-1.0009) | 0.347 | 0.241 |
|  |  | Weighted median |  | 0.46 | 1.0003  (0.9996-1.0010) |  |  |
|  |  | MR Egger |  | 0.25 | 1.0006  (0.9996-1.0016) |  |  |
|  | Peripheral atherosclerosis  (DM_PERIPHATHERO) | Inverse variance weighted | 26 | 0.005 | 1.0002 (1.0001-1.0003) | 0.519 | 0.346 |
|  |  | Weighted median |  | 0.01 | 1.0002  (1.0001-1.0003) |  |  |
|  |  | MR Egger |  | 0.01 | 1.0002  (1.0001-1.0004) |  |  |
|  | AF (ukb-b-964) | Inverse variance weighted | 30 | 0.74 | 1.0000  (0.9997-1.0002) | 0.689 | 0.291 |
|  |  | Weighted median |  | 0.76 | 0.9999  (0.9996-1.0003) |  |  |
|  |  | MR Egger |  | 0.60 | 0.9999  (0.995-1.0003) |  |  |
|  | Stroke (ukb-d-C_STROKE) | Inverse variance weighted | 39 | 0.16 | 1.0002  (0.9999-1.0006) | 0.106 | 0.149 |
|  |  | Weighted median |  | 0.09 | 1.0004  (0.9999-1.0008) |  |  |
|  |  | MR Egger |  | 0.00 | 1.0007  (1.0002-1.0012) |  |  |
|  | HF (ukb-d-HEARTFAIL) | Inverse variance weighted | 37 | 0.44 | 0.9999  (0.9998-1.0001) | 0.196 | 0.941 |
|  |  | Weighted median |  | 0.30 | 0.9999  (09997-1.0001) |  |  |
|  |  | MR Egger |  | 0.15 | 0.9998  (0.9996-1.0001) |  |  |
|  |  | Weighted median |  | 0.30 | 1.0003  (0.9997-1.0010) |  |  |
|  |  | MR Egger |  | 0.08 | 1.0007  (0.9999-1.0014) |  |  |
|  | CAD (ukb-d-I9_IHD) | Inverse variance weighted | 32 | 0.45 | 1.0003  (0.9996-1.0010) | 0.700 | 0.353 |
|  |  | Weighted median |  | 0.63 | 1.0002  (0.9994-1.0010) |  |  |
|  |  | MR Egger |  | 0.43 | 1.0004  (0.9994-1.0015) |  |  |
|  | MI (ukb-d-I9_MI) | Inverse variance weighted | 33 | 0.13 | 1.0003  (0.9999-1.0006) | 0.843 | 0.585 |
|  |  | Weighted median |  | 0.34 | 1.0002  (0.9998-1.0007) |  |  |
|  |  | MR Egger |  | 0.24 | 1.0003  (0.9998-1.0008) |  |  |

Notes: UK biobank data were extracted from the Neale lab analysis results (http://www.nealelab.is/uk-biobank), PAS, Peripheral atherosclerosis; CAS, Coronary atherosclerosis; MI, myocardial infraction; HF, heart failure; AF; atrial fibrillation; CAD, coronary artery disease.
